# Current Landscape of Targeted Therapies for Hormone-Receptor Positive, HER2 Negative Metastatic Breast Cancer

**DOI:** 10.3389/fonc.2018.00308

**Published:** 2018-08-10

**Authors:** Tarah J. Ballinger, Jason B. Meier, Valerie M. Jansen

**Affiliations:** ^1^Division of Hematology-Oncology, Department of Medicine, Indiana University School of Medicine, Indianapolis, IN, United States; ^2^Division of Hematology-Oncology, Department of Medicine, Vanderbilt University Medical Center, Nashville, TN, United States

**Keywords:** breast cancer, endocrine therapy, cyclin dependent kinase inhibitor, endocrine resistance, targeted therapy

## Abstract

The majority of deaths from MBC are in patients with hormone receptor (HR) positive, HER2 negative disease. Endocrine therapy (ET) remains the backbone of treatment in these cases, improving survival and quality of life. However, treatment can lose effectiveness due to primary or acquired endocrine resistance. Analysis of mechanisms of ET resistance has led to the development of a new generation of targeted therapies for advanced breast cancer. In addition to anti-estrogen therapy with selective estrogen receptor modulators, aromatase inhibitors, and/or selective estrogen receptor degraders, combinations with cyclin dependent kinase (CDK) 4/6 inhibitors have led to substantial progression free survival (PFS) improvements in the first and second line settings. While the PI3K/AKT/mTOR pathway is known to be an important growth pathway in HR positive breast cancer, PI3K inhibitors have been disappointing due to modest effect sizes and significant toxicity. The mTOR inhibitor everolimus significantly improves progression free survival when added to ET, and recent studies have improved supportive care allowing less toxicity. While these combination targeted therapies improve outcomes and often delay initiation of chemotherapy, long term overall survival data are lacking and data for the ideal strategy for sequencing these agents remains unclear. Ongoing research evaluating potential biomarkers and mechanisms of resistance is anticipated to continue to improve outcomes for patients with HR positive metastatic breast cancer. In this review, we will discuss management and ongoing challenges in the treatment of advanced HR positive, HER2 negative breast cancer, highlighting single agent and combination endocrine therapies, targeted therapies including palbociclib, ribociclib, abemaciclib, and everolimus, and sequencing of therapies in the clinic.

## Introduction

With approximately 250,000 new cases and 40,000 deaths annually, breast cancer is the most common malignancy and a leading cause of cancer-related death for women in the United States (1). The majority of breast cancers are hormone receptor positive (HR positive), including estrogen receptor (ER) and/or progesterone receptor (PR) positive, with endocrine therapy (ET) remaining the backbone of therapy (2–4). Approximately 5–10% of breast cancer patients present with *de novo* metastatic disease, and 30% of those initially treated with ET for early stage disease will relapse with metastatic spread (2). In the setting of metastatic breast cancer (MBC), the goal of treatment is palliative, and ET plays a crucial role of maintaining disease control, delaying the need for chemotherapy, and preserving quality of life. Multiple new agents have been developed to treat patients in the event of endocrine resistance; however, optimal sequencing of these therapies has not yet been determined in clinical trials. Herein, we present a review of the current treatment landscape for optimal management of HR positive, HER2 negative MBC.

## Endocrine therapies

ET includes the selective estrogen receptor modulators (SERMs; tamoxifen), aromatase inhibitors (AIs; letrozole, anastrozole, exemestane) and selective estrogen receptor down regulators (SERDs; fulvestrant). Given the heterogeneity of disease presentation and patient characteristics, there is no standard consensus recommendation for the optimal sequencing of these agents in patients with HR positive breast cancer in the metastatic setting (5–8). Results from several phase III studies evaluating single agent and combination endocrine therapy are detailed in Table 1.

**Table 1:** Summary of important phase III clinical trial results for endocrine therapy in metastatic HR positive, HER2 negative breast cancer.

**Line**	**Study**	**Design**	***n***	**ORR**	**PFS or TTP HR (95% CI)**	**OS (months) HR (if aval.)**
1st	North American Trial	Anastrozole 1 mg vs. tamoxifen 20 mg	353	21 vs. 17%	11.1 vs. 5.6 mo 1.44 (1.16)	40.4 vs. 38.5 1.02 (0.81-NR)
	ILBCG	Letrozole 2.5 mg vs. tamoxifen 20 mg	907	32 vs. 21%	9.4 vs. 6.0 mo 0.70 (0.60–0.82)	34 vs. 30
	EORTS	Exemestane 25 mg vs. tamoxifen 20 mg	371	46 vs. 31%	9.9 vs. 5.8 mo	37.2 vs. 43.3
	FIRST (phase II)	Fulvestrant 500 mg vs. anastrozole 1 mg	205	31.4 vs. 31.1	23.4 vs. 13.1 mo 0.66 (0.47–0.92)	54.1 vs. 48.4 0.70 (0.50–0.98)
	FACT	Fulvestrant 250 mg + anastrozole 1 mg vs. anastrozole 1 mg	514	31.8 vs. 33.6%	10.8 vs. 10.2 mo 0.99 (0.81–1.20)	37.8 vs. 38.2 1.0 (0.76–1.32)
	FALCON	Fulvestrant 500 mg vs. anastrozole 1 mg	562	46 vs. 45%	16.6 vs. 13.8 mo 0.80 (0.64-1.00)	Pending
	S0226	Fulvestrant 250 mg + anastrozole 1 mg vs. anastrozole 1 mg	707	27 vs. 22%	13.5 vs. 15.0 mo 0.80 (0.68-0.94)	41.3 vs. 47.7 0.81 (0.65–1.00)
2nd	SoFEA	Fulvestrant 250 mg + anastrozole or placebo vs. exemestane (PD on NSAI)	723	7 vs. 7 vs. 4%	4.4 vs. 4.8 vs. 3.4 mo	Not reported
	EFECT	Fulvestrant 250 mg vs. exemestane 25 (PD on NSAI)	693	7.4 vs. 6.7%	3.7 vs. 3.7 mo 0.93 (0.82–1.13)	Not reported
	CONFIRM	Fulvestrant 250 mg vs. fulvestrant 500 (PD on ET)	736	10.2 vs. 9.1%	6.5 vs. 5.5 mo 0.80 (0.68–0.94)	26.4 vs. 22.3 0.81 (0.69–0.96)

*ORR, overall response rate; PFS, progression free survival; TTP, time of progression; HR, hazard ratio; OS, overall survival; NSAI, nonsteroidal aromatase inhibitor; ET, endocrine therapy*.

One of the oldest of these therapies is the SERM tamoxifen, approved by the FDA as adjuvant therapy in 1986 and repeatedly found to be effective in the metastatic setting. A systematic review of 86 clinical trials showed an overall response rate (ORR) of 34% for single agent tamoxifen (9). In pre-menopausal patients with MBC, first-line therapy with ovarian ablation with either goserelin or buserelin and tamoxifen was associated with a significant increase in OS when compared to ovarian ablation alone (HR 0.78, 95% CI 0.63–0.96, *p* = 0.02), with a significant improvement in PFS as well (HR 0.70, 95% CI 0.59–0.85) (10). For post-menopausal patients, in whom estrogen synthesis occurs in the peripheral tissues through aromatization, treatment with AIs has largely replaced tamoxifen. There is no clinical evidence to suggest favoring one AI over another. Although there are mechanistic differences between the steroidal AI (exemestane) and non-steroidal AIs (letrozole and anastrozole) (11), they are not fully cross-resistant (12). AIs have been associated with improved overall response rate (ORR), time to progression (TTP) and disease control rate when compared to tamoxifen (13). A large meta-analysis demonstrated superior survival for patients receiving AI compared to tamoxifen (HR 0.89, 95% CI 0.80–0.99) (9). Side effect profiles of AIs and SERMs vary, with AIs increasing rates of bone loss and musculoskeletal side effects, and tamoxifen carrying an increased risk of thromboembolic disease and uterine cancer (14).

Treatment with SERDs represents another viable option for ET. Initial studies of fulvestrant 250 mg showed modest anti-tumor activity, while higher dosing with 500 mg has been found to provide superior disease control and a greater survival advantage in comparison to AIs (15–18). In the FIRST study of 205 post-menopausal patients with advanced breast cancer, fulvestrant 500 mg was found to have a superior TTP and a decreased risk of progression when compared to anastrozole (19). Subsequent analysis of the FALCON trial comparing fulvestrant 500 mg and anastrozole in the first-line metastatic setting found that fulvestrant was associated with a significant improvement in PFS (16.6 months vs. 13.8 months, *p* = 0.048), with similar rates of adverse events such as arthralgias and hot flashes (20). Of note, most of the patients in the FALCON study had never received prior endocrine therapy. While fulvestrant alone remains an option for endocrine naïve patients in the first line setting, single agent response rates are low in previously treated patients (21) and the majority of patients will now receive a CDK4/6 inhibitor, as discussed later in this review.

## Combination endocrine therapy

Combination therapy with anti-endocrine agents is a logical extension of single agent anti-endocrine therapy, given the mechanistic differences between the agents. Based on encouraging preclinical studies, combination treatment with fulvestrant plus anastrozole was compared to single agent anastrozole in the FACT and SWOG S0226 trials. Of note, both trials used a lower fulvestrant dosing schedule (500 mg loading, then 250 mg monthly). In the FACT trial, combination endocrine therapy did not demonstrate any significant improvement in TTP or OS (22). In the SWOG S0226 trial, combination therapy was associated with an improved PFS, [13.5 months vs. 15.0 months (HR 0.8 95% CI 0.68–0.94; *p* = 0.007)] and OS [41.3 months vs. 47.7 months (HR 0.81 95% CI 0.65–1.00; *p* = 0.05)] (23). The difference in these results likely has to do with differing populations in each study. While the SWOG S0226 trial participants were largely treatment naive, approximately two thirds of participants in the FACT trial had received previous anti-estrogen therapy.

Given the lack of benefit for combination anti-endocrine therapy seen in the FACT trial, the South Korean SoFEA trial was explicitly designed to address the population of MBC patients with progression on prior non-steroidal AI therapy. In a randomized three arm trial, combination therapy with fulvestrant (500 mg loading dose and 250 mg monthly) plus anastrozole was compared to fulvestrant plus placebo or single agent exemestane. No significant differences were identified between the combination arm and either of the single agent arms for PFS or OS (24). A subsequent Cochrane Review of fulvestrant in MBC incorporating 4,514 patients found no benefit for fulvestrant when used in combination ET in the first or second line settings (25).

## Mechanisms of endocrine resistance

A major obstacle in the treatment of HR positive MBC is intrinsic or acquired resistance to ET. There are multiple oncogenic drivers and resistance pathways in HR positive breast cancer, and thorough reviews of these complicated mechanisms can be found in the literature elsewhere (26–28). Relevant to this review, several resistance mechanisms have led to the development of therapeutic strategies, including dysregulation of cell cycle progression, activation of the PI3K/AKT/mammalian target of rapamycin (mTOR) pathway, and *ESR1* mutations.

Upregulation of cell cycle regulating molecules, especially those involved in progression of cells from the G1 to S phases, can contribute to endocrine resistance (26). Preclinical work identified CDK4 as critical for growth of ER positive breast cancer cells resistant to estrogen deprivation (29). Further, *in vitro* models of fulvestrant resistant breast cancers revealed increased expression of CDK6 when compared to fulvestrant sensitive cells. CDK4/6 is activated when bound to cyclin D1 in the mid-to-late G1 phase of the cell cycle. CDK4/6 phosphorylates Rb to inactivate its repressive hold on E2F-mediated cell cycle progression (30, 31). CDK4/6 activity is particularly relevant in ER positive breast cancers, given that the cyclin D1 gene *CCND1* is regulated by ERα (32). Laboratory and clinical studies confirm that CDK4/6 inhibitors potently decrease growth of ER positive breast cancers (33–35). Biomarker analysis of 91 patients with resistance to fulvestrant demonstrated that elevated CDK6 levels associated with a shorter PFS (36).

Molecularly, estrogen activity can induce activation of insulin-like growth factor and the PI3K/AKT and MAPK (mitogen activated protein kinase) pathways, which can downregulate ER and PR cell surface expression (37–41). Increased PI3K/AKT signaling can also result in hormone receptor deactivation, and promote ligand independent growth of malignancies in the absence of endocrine signaling. PI3K/AKT signaling has also been demonstrated to directly phosphorylate the estrogen receptor, resulting in estrogen independent activation of hormone receptors and providing a secondary mechanism of ET resistance (42). PTEN (phosphatase and tensin homolog) is a negative regulator of the PI3K pathway, and PTEN expression is downregulated in the setting of endocrine resistance (43).

Multiple genomic alterations have been identified in MBCs with both intrinsic and acquired resistance, including in *ESR1*, resulting in constitutive activation of ER. In correlative analysis of several trials, frequency of *ESR1* mutations in circulating tumor DNA (ctDNA) is between 25 and 39% (44–46). *ESR1* mutations have been linked to poorer outcomes in patients treated with AI based therapies. In a secondary analysis of the BOLERO-2 trial of exemestane with or without the mTOR inhibitor everolimus, the presence of an *ESR1* mutation was associated with reduced OS (47). Data have suggested that SERDs, such as fulvestrant, may overcome *ESR* mutations. In the SoFEA study, patients the *ESR1* mutated tumors had significantly worse outcomes when treated with exemestane, but not when treated with fulvestrant (HR 0.52, 95% CI 0.30–0.92, *p* = 0.02) (44).

Given the effectiveness of ET in the HR positive population, therapies designed to overcome *de novo* or acquired resistance targeting the aforementioned signaling and cell cycle regulating molecules may be particularly effective at extending OS and PFS in this population. In the next section, we review the small molecule inhibitors that have been approved or are being investigated in combination with antiestrogens for the treatment of post-menopausal women with advanced HR positive, HER2 negative breast cancer in the first or later line settings, targeting both intrinsic and acquired resistance.

### CDK4/6 inhibitors

#### Palbociclib

Palbociclib was originally reported in 2009 as PD032991 and noted to selectively inhibit cyclin-dependent kinases 4 and 6, which resulted in selective inhibition of the proliferation of luminal ER positive breast cancer cell lines, while non-luminal basal cell lines were largely resistant to treatment. Molecular analysis of these cell lines revealed that phosphorylated retinoblastoma (Rb) and cyclin D1 were elevated while p16 was decreased in sensitive luminal cell lines. This Rb phosphorylation was inhibited by treatment with palbociclib resulting in G0/G1 cell cycle arrest (48). It is important to note that while the anti-proliferative effects of palbociclib were synergistic with anti-estrogen and anti-HER2 therapy in sensitive cell lines (48), in triple negative breast cancer and RB1-proficient cell lines, palbociclib was actually antagonistic to anthracycline mediated cytotoxicity (49). The dose limiting toxicity of palbociclib is neutropenia resulting from cell cycle arrest of marrow hematopoietic stem cells and precursors. This is reversible upon discontinuation of the drug, in contrast to chemotherapeutic agents such as paclitaxel or doxorubicin (50).

PALOMA-1/TRIO-18, a phase II study, assessed the safety and efficacy of palbociclib in conjunction with letrozole as first line treatment of HR positive, HER2 negative advanced breast cancer. The study examined two cohorts, enrolling the first based on receptor status, while the second was enrolled afterwards with additional requirements for amplification of cyclin D1, loss of p16, or both. Both cohorts were randomized (1:1) to receive letrozole 2.5 mg daily with or without oral palbociclib 125 mg daily for 21 days out of a 28-day cycle. The primary endpoint of investigator determined PFS was found to be 10.2 months for letrozole alone, compared to 20.2 months for palbociclib plus letrozole (HR 0.49, 95% CI 0.32–0.75, *p* = 0.0004). In cohort 1, PFS was 26.1 months for the palbociclib plus letrozole group, vs. 5.7 months for letrozole alone (HR 0.30, 95% CI 0.16–0.57, *p* < 0.0001). In the second cohort with potential biomarkers, PFS was 18.1 months for the palbociclib plus letrozole group, vs. 11.1 months with letrozole alone (HR 0.51, 95% CI 0.30–0.85, *p* = 0.0046). Accrual to cohort 2 was stopped early due to unplanned interim analysis of cohort 1 and amendment of planned combined cohort analysis. The different outcomes between cohorts 1 and 2 suggested a different behavior of the CCND1 amplified or p16 deficient tumors, and support ER positivity as the most important biomarker for response to AI plus CDK4/6 inhibition, with additional molecular markers of response remaining elusive, as discussed below. In the toxicity analysis, grade 3–4 side effects were more common in the palbociclib group, with neutropenia (54 vs. 1%) leukopenia (19 vs. 1%) and fatigue (4 vs. 1%) being the most common (33). Based on the results of the PALOMA-1/TRIO 18 study, palbociclib received accelerated approval in combination with letrozole for first line therapy for post-menopausal women with advanced HR positive, HER2 negative breast cancer and subsequent therapy after progression on prior endocrine therapy (51). The follow up phase III study, PALOMA-2, confirmed these results, leading to regular approval of palbociclib (52).

In PALOMA-3, a double-blind phase III study to assess the efficacy and safety of palbociclib plus fulvestrant in ET resistant (define as progression on ET or relapse within 12 months of adjuvant ET) HR positive metastatic breast cancer, the combination of palbociclib plus fulvestrant was associated with significant improvement in PFS compared to fulvestrant and placebo. Median PFS was 9.5 months in the fulvestrant plus palbociclib group, compared to 4.6 months in the fulvestrant plus placebo group (HR 0.46, 95% CI 0.36–0.59, *p* < 0.0001) (53). Analysis of patient reported outcomes revealed that treatment with palbociclib plus fulvestrant resulted in higher global quality of life scores and significant reduction in pain when compared to fulvestrant alone (54).

#### Ribociclib

Ribociclib, originally designated LEE011, is an oral small molecule inhibitor of CDK4/6 similar to palbociclib (55). In preclinical models, ribociclib was found to be highly selective for CDK4/6 and function primarily with G1 arrest and regression in some animal models (56). Ribociclib was approved based up on the MONALEESA-2 study, a phase III randomized, double-blind trial of 668 patients evaluating ribociclib plus letrozole for first line treatment of women with HR positive, HER2 negative MBC. The trial was stopped early after a preplanned interim analysis revealed a significant advantage for the ribociclib containing arm. With a median follow up of 15.3 months, the PFS was significantly longer in the ribociclib containing arm (HR 0.56; 95% CI, 0.43–0.72; *p* < 0.001). Updated analyses show a median PFS of 25.3 months with ribociclib vs. 16.0 months for placebo (57). Common grade 3/4 adverse events in the ribociclib group were neutropenia (59.3 vs. 0.9%) and leukopenia (21.0 vs. 0.6%) (35). MONALEESA-3 is a phase III study of fulvestrant in combination with ribociclib or placebo for both first and second line treatment of HR positive, HER2 negative MBC, with results reported at the 2018 American Society of Clinical Oncology annual meeting. Consistent with other trials, PFS was significantly improved in both the first line (HR 0.577, 95% CI 0.42–0.80) and second line/early relapse settings (HR 0.565, 95% CI 0.43–0.74) (58).

MONALEESA-7 specifically addressed the pre-menopausal group. Presented at the 2017 San Antonio Breast Cancer Symposium (SABCS), this phase III trial compared ribociclib vs. placebo in pre-menopausal woman receiving an AI or tamoxifen with ovarian suppression. Median PFS again favored the ribociclib arm (23.8 vs. 13.0 months, HR 0.55, 95% CI 0.44–0.69, *p* < 0.0001) (59).

#### Abemaciclib

Abemaciclib is the third agent in the class of CDK4/6 inhibitors that is currently FDA approved.

MONARCH-2, a global, double-blind phase III trial investigating abemaciclib in combination with fulvestrant compared to fulvestrant alone in HR positive, HER2 negative MBC with progression on prior ET, demonstrated an improved PFS with the combination therapy (16.4 vs. 9.3 months, HR 0.553, 95% CI 0.449–0.681, *p* < 0.001). Treatment with abemaciclib was associated with increased rates of diarrhea (86.4 vs. 24.7%), neutropenia (46.0 vs. 4.0%), nausea (45.1 vs. 22.9%), and fatigue (39.9 vs. 26.9%), as well as an increased rate of thromboembolic events (2.0 vs. 0.4%) (60). Results of the phase III study of abemaciclib in combination with a non-steroidal AI (MONARCH-3) were recently reported (61). This study allowed the treating physician to determine the non-steroidal AI utilized (79.1% letrozole, 20.9% anastrozole). Interim analysis was conducted following 194 PFS events with a median follow up of 17.8 months. Abemaciclib was associated with improved PFS (14.7 months vs. not reached, HR 0.543, 95% CI 0.41–0.72; *p* < 0.001).

MONARCH-1, a phase II single arm study, evaluated the safety and efficacy of abemaciclib monotherapy in patients with HR positive, HER2 negative metastatic breast cancer of any menopausal status, whose disease had progressed on prior endocrine and chemotherapy. At the time of interim analysis 132 patients had been treated with abemaciclib 200 mg twice daily as monotherapy, with a median of three lines of prior therapy and over 90% having visceral disease. At 12 months, the ORR was 19.7%, median PFS of 6.0 months, and median OS of 17.7 months (62).

#### Comparative efficacy, sequencing, and biomarkers

Palbociclib, ribociclib, and abemaciclib have never been directly compared and have resulted in similar effect sizes (see Table 2). Differences in median PFS are attributable to differing patient populations between studies, with similar hazard ratios in studies across the three approved CDK4/6 inhibitors. After the 2018 ASCO annual meeting, we have results for all of these agents both in the first line setting with AIs and the second line setting in combination with fulvestrant. In addition, MONALESSA-7 and analyses from PALOMA-3 and MONARCH-2 have shown similar efficacy for CDK4/6 inhibitors in combination with ET and ovarian suppression for pre-menopausal women, confirming what many oncologists were already doing in practice (59, 63).

**Table 2:** Summary of phase III clinical trial results for CDK4/6 inhibitors in metastatic HR positive, HER2 negative breast cancer.

**Line**	**Study**	**Investigational arm**	***n***	**ORR**	**Median PFS HR (95% CI)**	**OS**
1st	PALOMA-2	Palbociclib/letrozole	666	55 vs. 39.0%	24.8 vs. 14.5 mo 0.58 (0.46–0.72)	Pending
	MONALEESA-2	Ribociclib/letrozole	668	54.5 vs. 38.8%	25.3 vs. 16.0 mo 0.57 (0.46–0.70)	Pending
	MONARCH-3	Abemaciclib/NSAI	493	59.0 vs. 44.0%	28.1 vs. 14.7 mo 0.54 (0.41–0.72)	Pending
	MONALEESA-7	RIbociclib/AI or tam+OFS	672	51.0 vs. 36.0%	23.8 vs. 13.0 mo 0.55 (0.44–0.69)	Pending
2nd	PALOMA-3	Palbociclib/Fulvestrant	521	24.6 vs. 10.9%	9.5 vs. 4.6 mo 0.46 (0.36–0.59)	Not yet reported
	MONARCH-2	Abemaciclib/fulvestrant	669	48.1 vs. 21.3%	16.4 vs. 9.3 mo 0.55 (0.45–0.68)	Pending
	MONALEESA-3	RIbociclib/fulvestrant	726	41.0 vs. 29.0%	20.5 vs. 12.8 mo 0.59 (0.48–0.73)	Pending
Later	MONARCH-1^*^ Phase II	Abemaciclib (single arm)	132	19.7%	6.0 mo	17.7 mo

*ORR, overall response rate; PFS, progression free survival; HR, hazard ratio; OS, overall survival; NSAI, nonsteroidal aromatase inhibitor*.

While the three agents likely have similar efficacy, the toxicity profiles, dosing schedules, and monitoring required differ (64). Palbociclib and ribociclib, given on a 3 weeks on and 1 week off schedule, both have dose limiting toxicity of neutropenia, while the dose limiting toxicity for daily abemaciclib is diarrhea, perhaps due to its greater affinity for CDK4 over CDK6. Complete blood count (CBC) monitoring is required for all agents. Ribociclib uniquely requires monitoring of liver function tests, electrolytes, and the QT interval. Abemaciclib requires liver function test monitoring as well, and may increase risk of thromboembolic disease. There is no specific benefit of one toxicity profile over another, and patient comorbidities, preferences, and physician familiarity dictate choice between these agents. Packaging provided by drug companies also differs between the agents, with the blister packaging of ribociclib potentially allowing for dose reduction in a more timely manner without the need for a new prescription. Importantly, the financial toxicity of each of these agents can be significant.

While the data for the use of CDK4/6 inhibitors in the first or second line has been impressive, questions remain. Overall survival data from the larger, randomized trials remains to be seen and will likely be difficult to interpret given the long natural history of HR positive MBC. In addition, data clarifying when it is best to sequence a CDK4/6 inhibitor in the course of a patient's therapy options are lacking. Regardless, PFS, quality of life, and delay of chemotherapy are important clinical benefits in the absence of survival data, and it is likely that receiving a CDK4/6 inhibitor at some point is better than never having received one. The decision of use in the first or second line should be based on disease characteristics and patient preference. While the majority of patients will likely receive a CDK4/6 inhibitor in the first line setting, select patients with a long disease-free interval after adjuvant ET, or small volume or bone-only disease, are likely to have a long PFS interval with ET therapy alone and may opt to delay CDK4/6 inhibitor initiation to a later line.

Despite the promising results, approximately 20% of patients will not respond to CDK4/6 inhibitors and the majority will develop resistance. Thus far, there has been no subgroup or clinical population that does not derive benefit from CDK4/6 inhibitors, including age, menopausal status, visceral vs. bone metastases, race, or treatment free interval. In addition, an FDA pooled analysis looking at rarer clinical subtypes, including lobular disease, PR negative, or *de novo* MBC, found continued benefit to all subtypes (65). It is important to note that the CDK4/6 inhibitors have high and potentially rapid response rates in patients with even advanced visceral disease, and can therefore be used instead of chemotherapy in the absence of true “visceral crisis.”

In addition to clinical subtypes, there has been a consistent benefit to CDK4/6 inhibitors across molecular biomarkers thought to be involved in resistance, including pRb, p16, Ki67, *ESR1* expression, *CDKN2A* expression, *CCND1* expression, *PI3KCA* mutations, and *ESR1* mutations, among others. An abstract presented at the 2018 ASCO annual meeting looking at mRNA expression in MONALESSA-2 found a trend toward longer PFS with higher *ESR1* expression, and a trend toward more benefit from ribociclib with high *ESR1* expression, compared to low (HR 0.39 vs. HR 0.74) (66). Mechanisms of resistance to CDK4/6 inhibitors are reviewed elsewhere and thought to include cyclin E amplification, Rb loss, *CDK2* overexpression, *FGFR1* amplification or mutation, and upregulation of the PI3k/AKT pathway (67, 68). These investigations have led to the development of clinical trials assessing drug combinations, including CDK4/6 inhibition with PI3K or mTOR inhibitors.

### PI3K/AKT/mTOR inhibitors

#### mTOR inhibitors

mTOR is a PI3K kinase with roles in the regulation of PI3K signaling and regulation of protein synthesis and cell growth (69). In breast cancer, activated mTOR signaling is associated with poor patient survival (70) and worse prognosis (71). mTOR can be activated by activating mutations in the *PIK3CA* gene (72), alteration or mutation of AKT, or loss of PTEN signaling (73). mTOR activation has been associated with resistance to SERMs and AIs (74, 75) and inhibition of the mTOR pathway is associated with resensitization of breast cancer cells to hormonal therapy (76). In breast cancer cells resistant to AI, treatment with the mTOR inhibitor everolimus has been associated with dramatically reduced ER expression and increased autophagy in MCF-7 breast cancer cell lines (77, 78). Results from phase III trials assessing agents in this pathway are summarized in Table 3.

**Table 3:** Summary of phase III clinical trial results for PI3K and mTOR inhibitors in metastatic HR positive, HER2 negative breast cancer.

**Line**	**Study**	**Investigational arm**	***n***	**ORR**	**Median PFS HR (95% CI)**	**OS**
2nd	BOLERO-2	Everolimus/exemestane	724	9.5 vs. 0.4%	6.9 vs. 2.8 mo 0.43 (0.35–0.54)	31.0 vs. 26.6 mo 0.89 (0.73–1.1)
	BELLE-2	Buparlisib/fulvestrant	1,147	11.8 vs. 7.7%	6.9 vs. 5.0 mo 0.78 (0.67–0.89)	Pending
	BELLE-3	Buparlisib/fulvestrant	432	22 vs. 3%	3.9 vs. 1.8 mo 0.67 (0.53–0.84)	Pending
	SANDPIPER	Talelisib/fulvestrant	516 Pi3K mutants	28 vs. 11.9%	7.4 vs. 5.4 mo 0.70 (0.56–0.89)	Pending

*ORR, overall response rate; PFS, progression free survival; HR, hazard ratio; OS, overall survival*.

Based on preclinical and neoadjuvant data, the combination of everolimus plus exemestane was assessed for efficacy and safety in patients with HR positive, HER2 negative breast cancer refractory to non-steroidal AIs in the international double-blind phase III BOLERO-2 study. The primary end point of PFS was met by the study, with the advantage for the combination arm (6.9 vs. 2.8 months, HR 0.43, 95% CI 0.35–0.54, *p* < 0.001). Twenty-three percent of patients treated with everolimus reported serious adverse events, and 19% of patients (4% treated with placebo) discontinued therapy due to adverse events. The most common grade 3/4 toxicities were stomatitis (8%), anemia (6%), dyspnea (4%), and pneumonitis (3%) (79). Further analysis in the BALLET study of everolimus and exemestane in patients previously treated with or without chemotherapy, found a high rate of adverse events, the most common being stomatitis (51.3%) and confirmed the safety profile was not affected by prior chemotherapy (80). In addition, despite the increased toxicity, there was no evidence of statistically significant improvement in OS (hazard ratio 0.89; 95% CI 0.73–1.10; *P* = 0.14) (81).

As stomatitis has been a consistent problem in completing everolimus therapy, the phase II SWISH study investigated the use of prophylactic steroid mouthwash. Of the 85 women evaluable for efficacy, only 2% had grade 2 or worse stomatitis, vs. 33% historically in BOLERO-2 (82). A similar phase III trial (Oral Care-BC) is investigating the impact of professional dental cleanings by oral surgeons on the incidence of oral mucositis with everolimus therapy. The study is still accruing with estimated primary completion date of March 2019 (NCT02376985) (83).

Vistusertib, an oral dual inhibitor of mTORC1 and mTORC2, was superior to everolimus (an mTORC1 inhibitor) in preclinical models and therefore was investigated in the phase II randomized study MANTA, evaluating fulvestrant alone versus fulvestrant with daily vistusertib, fulvestrant with intermittent vistusertib, and fulvestrant with everolimus. No significant difference was seen between the arms, with the exception of improved PFS in the fulvestrant with everolimus arm compared to fulvestrant with vistusertib (HR 0.64, 95% CI 0.45–0.91, *p* = 0.01), and fulvestrant with everolimus compared to fulvestrant alone (HR 0.64, 95% CI 0.43–0.94, *p* = 0.02) (84).

#### PI3K inhibitors

PI3K/AKT signaling plays an important role in the growth and proliferation of tumor cells by acting upstream of the mTOR signaling pathway. This signaling pathway is tightly regulated by the tumor repressor genes PTEN and INPP4B, which are frequently downregulated in breast malignancies, resulting in amplification of the PI3K/AKT/mTOR signal (85). *PIK3CA* mutations are also frequently mutated in HR positive breast cancer and are frequently associated with the luminal B subtype of breast cancer (86). Although *PIK3CA* mutations are frequently detected in breast cancers [32% in TCGA (87–89)], they have not been associated with clinical outcome (87) or influenced OS (90). However, in multivariate analysis, *PIK3CA* mutation in conjunction with node positive disease has been associated with unfavorable OS (87).

There are currently no PI3K inhibitors approved for HR positive breast cancer, although several are in registration trials. The most heavily studied has been buparlisib (BKM120), an oral pan-PI3K inhibitor under examination in the BELLE-2 (NCT01610284) and BELLE-3 (NCT01633060) phase III clinical trials in combination with fulvestrant in HR positive, HER2 negative, locally advanced, or MBC refractory to AI or AI+mTOR inhibitor, respectively. In BELLE-2, initial PFS data shows a significant advantage for the buparlisib arm (6.9 vs. 5.0 months, HR 0.78, 95% CI 0.67–0.89, *p* < 0.001). In BELLE-3, similar results were seen favoring the buparlisib arm, but PFS was short (3.9 vs. 1.8 months, HR 0.67, 95% CI 0.53–0.84), *p* < 0.001). On retrospective analysis of ctDNA, the benefit was restricted to the *PIK3CA* mutated group, while this was not seen in BELLE-3. In addition, significant side effects were seen, including elevated liver function tests, hyperglycemia, anxiety, and depression, resulting in dose delay and interruption (91, 92).

Taselisib (GDC-0032), a selective PI3K inhibitor with greater *in vitro* activity against mutant isoforms, has been studied in the phase III SANDPIPER trial, with initial results recently reported. Patients with *PIK3CA* mutated tumors with progression on prior AI were randomized 2:1 to taselisib with fulvestrant or placebo with fulvestrant. An exploratory arm enrolled patients with wild type tumors. In the mutated arms, PFS was improved with the addition of taselisib (7.4 vs. 5.4 months, HR 0.70, *p* = 0.0037). In the exploratory wild type group, there was no difference between the arms, but sample size was small. Fifty percent of patients in the investigational arm developed grade 3 or 4 toxicity, and the majority of patients discontinued treatment due to adverse events, diarrhea being most common (93).

While clinical trials have shown proof of concept for PI3K inhibition in endocrine resistance, the modest efficacy, significant toxicity, and unclear use within the CDK4/6 inhibitor spectrum will likely limit future development of these compounds.

In addition to PI3K inhibitors, several other classes of agents are under investigation to combat endocrine resistance but have yet to provide phase III clinical trial results. Several FGFR inhibitors are under investigation both in combination with endocrine therapy and with other targeted therapies (94). The oral histone deacetylase (HDAC) inhibitor entinostat in combination with exemestane improved DFS and OS over exemestane alone in a phase II trial (95) and the randomized phase III study is ongoing (NCT02115282).

## Conclusions

HR positive breast cancer is responsible for the majority of breast cancer related deaths. Although outcomes for HR positive breast cancer are excellent in early disease, in the advanced setting, outcomes remain poor with more women dying from HR positive breast cancer than other subtypes combined. In an era of personalized medicine with four small molecule inhibitors already FDA-approved (everolimus, pabociclib, riboclicib, abemaciclib), in addition to others under clinical investigation, the question of how to tailor therapy for patients in this setting is very relevant. Currently there is a lack of definitive biomarkers to identify specific patient populations that will benefit more from one targeted therapy over another.

Without clear biomarkers, selection of ET in the first line metastatic setting depends on clinician/patient preferences, disease burden, and disease biology. While endocrine therapy alone, including aromatase inhibitors, tamoxifen, or fulvestrant, may be appropriate for select patients, the consistent benefits of CDK4/6 inhibitors across multiple subgroups, including those with longer disease free intervals, support their use in the first line for most patients while we await overall survival data. Options for the ET backbone include AIs or fulvestrant for post-menopausal patients, and tamoxifen, AI, or fulvestrant with ovarian suppression for pre-menopausal patients. For most patients, AI (with or without ovarian suppression) and a CDK4/6 inhibitor will likely be most appropriate, with fulvestrant plus CDK4/6 inhibitor used in those who relapsed while on or within 12 months of adjuvant AI therapy, or those with a known *ESR1* mutation. Again, individual patient comorbidities, preferences for drug administration, and access should play a role in selection of first line therapy.

In the event of progression, second line treatment should include a CDK4/6 inhibitor if not used in the first line setting. While we await data on the utility of continued CDK4/6 inhibition or switching agents after progression, options for patients after progression include fulvestrant or an AI alone if not previously used, or exemestane with everolimus. We are in favor of enrollment in clinical trials for patients with CDK4/6 inhibitor resistance, including, but not limited to, the MAINTAIN trial evaluating fulvestrant with or without ribociclib after progression on palbociclib or ribociclib (NCT02632045); PACE evaluating fulvestrant, fulvestrant with palbociclib, or fulvestrant with palbociclib plus the anti-PD-1 avelumab in patients progressing on palbociclib (NCT03147287), or TRINITI, evaluating ribociclib with everolimus and exemestane after prior CDK4/6 inhibitor treatment (NCT02732119). For patients progressing rapidly through ET, standard cytotoxic chemotherapy can be tried.

Continued work to evaluate biomarkers of resistance may lead us in the future to a precision medicine approach to therapy after CDK4/6 inhibitor resistance develops. Recent data evaluating ctDNA before and after CD4/6 inhibitor treatment in PALOMA-3 showed the development of Rb, PIK3CA, and ESR1 mutations (96). This study indicated feasibility of detecting genomic changes in the blood, which might guide us to alter therapy in a targeted way. Correlative studies from the ongoing trials mentioned above will likely begin to help us answer these questions.

Single agent endocrine therapy and combinations with targeted therapy offer a range of options for patients with metastatic HR positive breast cancer. Currently, the optimal sequencing of these therapies is unclear, and should be based on patient characteristics, clinical judgement, and disease biology. While sequential lines of ET combinations can improve outcomes for patients, many acquire endocrine resistance. Ongoing research to identify biomarkers and optimal sequencing for individual patients is awaited, with hopes to continue to improve outcomes and quality of life for patients with HR positive metastatic breast cancer.

## Author contributions

TB and JM are responsible for the content of this article. TB and VJ are responsible for the intellectual content, analysis, and revision of the manuscript.

### Conflict of interest statement

The authors declare that the research was conducted in the absence of any commercial or financial relationships that could be construed as a potential conflict of interest.
